# Comparative analysis of targeted next-generation sequencing for *Plasmodium falciparum* drug resistance markers

**DOI:** 10.1038/s41598-022-09474-5

**Published:** 2022-04-01

**Authors:** Chanon Kunasol, Arjen M. Dondorp, Elizabeth M. Batty, Vorthunju Nakhonsri, Puritat Sinjanakhom, Nicholas P. J. Day, Mallika Imwong

**Affiliations:** 1 Department of Molecular Tropical Medicine and Genetics, Faculty of Tropical Medicine, Mahidol University, 420/6 Rajvithi Rd., Bangkok, 10400 Thailand; 2 Mahidol-Oxford Tropical Medicine Research Unit, Faculty of Tropical Medicine, Mahidol University, Bangkok, Thailand; 3 Centre for Tropical Medicine and Global Health, Nuffield Department of Medicine, University of Oxford, Oxford, UK; 4 National Biobank of Thailand (NBT), National Science and Technology Development Agency (NSTDA), 144 Innovation Cluster 2 Building (INC) Tower A, Thailand Science Park, Khlong Nueng, Khlong Luang District, Pathum Thani Thailand

**Keywords:** Biological techniques, Molecular medicine

## Abstract

Well-defined molecular resistance markers are available for a range of antimalarial drugs, and molecular surveillance is increasingly important for monitoring antimalarial drug resistance. Different genotyping platforms are available, but these have not been compared in detail. We compared Targeted Amplicon Deep sequencing (TADs) using Ion Torrent PGM with Illumina MiSeq for the typing of antimalarial drug resistance genes. We developed and validated protocols to type the molecular resistance markers *pfcrt*, *pfdhfr*, *pfdhps*, *pfmdr1*, *pfkelch*, and *pfcytochrome b*, in *Plasmodium falciparum* for the Ion Torrent PGM and Illumina MiSeq sequencing platforms. With *P. falciparum* 3D7 and K1 as reference strains, whole blood samples (N = 20) and blood spots from Rapid Diagnostic Test (RDT) samples (N = 5) from patients with uncomplicated falciparum malaria from Ubon Ratchathani were assessed on both platforms and compared for coverage (average reads per amplicon), sequencing accuracy, variant accuracy, false positive rate, false negative rate, and alternative allele detection, with conventional Sanger sequencing as the reference method for SNP calling. Both whole blood and RDT samples could be successfully sequenced using the Ion Torrent PGM and Illumina MiSeq platforms. Coverage of reads per amplicon was higher with Illumina MiSeq (28,886 reads) than with Ion Torrent PGM (1754 reads). In laboratory generated artificial mixed infections, the two platforms could detect the minor allele down to 1% density at 500X coverage. SNPs calls from both platforms were in complete agreement with conventional Sanger sequencing. The methods can be multiplexed with up to 96 samples per run, which reduces cost by 86% compared to conventional Sanger sequencing. Both platforms, using the developed TAD protocols, provide an accurate method for molecular surveillance of drug resistance markers in *P. falciparum*, but Illumina MiSeq provides higher coverage than Ion Torrent PGM.

## Introduction

The availability of efficacious antimalarial drug is pivotal for malaria control and elimination. Artemisinin-based combination therapies (ACT) are currently the first line drugs for uncomplicated falciparum malaria in all malaria endemic countries, and new antimalarial compounds are not expected within the next 5 years. Unfortunately, artemisinin resistance has emerged in Southeast Asia and more recently in Sub-Saharan Africa. In the Greater Mekong Subregion of Southeast Asia this has been compounded by ACT partner drug resistance, resulting in a dramatic loss of efficacy for several ACTs. Monitoring of the emergence and spread of antimalarial drug resistance is crucial for defining national malaria treatment guidelines. Clinical therapeutic efficacy studies are the reference method for monitoring drug resistance, but in-vitro drug efficacy testing and assessment of molecular resistance markers in the parasite gene are important additional tools^1^. The arsenal of molecular markers is constantly increasing. Reliable genetic markers are now available for resistance against chloroquine (chloroquine resistance transporter, *pfcrt*), antifolate drugs (dihydrofolate reductase, *pfdhfr*), sulfonamides (dihydropteroate synthase, *pfdhps*), mefloquine (multidrug drug resistance 1, *pfmdr1*), artemisinins (kelch propeller region, *pfkelch)*, piperaquine (plasmepsin II/III, *pfpmII/III*), and atovaquone (cytochrome B, *pfcytb*).

Next generation sequencing (NGS) using short reads is an increasingly used method to identify and track molecular markers for antimalarial drug resistance markers^2–10^. Two platforms, Ion Torrent PGM platform and Illumina MiSeq platform, are widely used, and are based on different principles. Ion Torrent PGM platform uses a semiconductor method to detect the proton that is released from the nucleotide incorporated during synthesis^11^, whereas Illumina platform uses fluorescently labeled reversible-terminator nucleotides incorporated in clonal amplified DNA captured in a flow cell^12^. These NGS techniques require only a small volume of DNA template, and are suitable for multiplexing hundreds of samples including different genetic markers in a single reaction run. In addition, NGS is highly accurate, fast and likely more cost-effective compared to conventional genotyping methods^2,4,13^.

**Figure 1:**
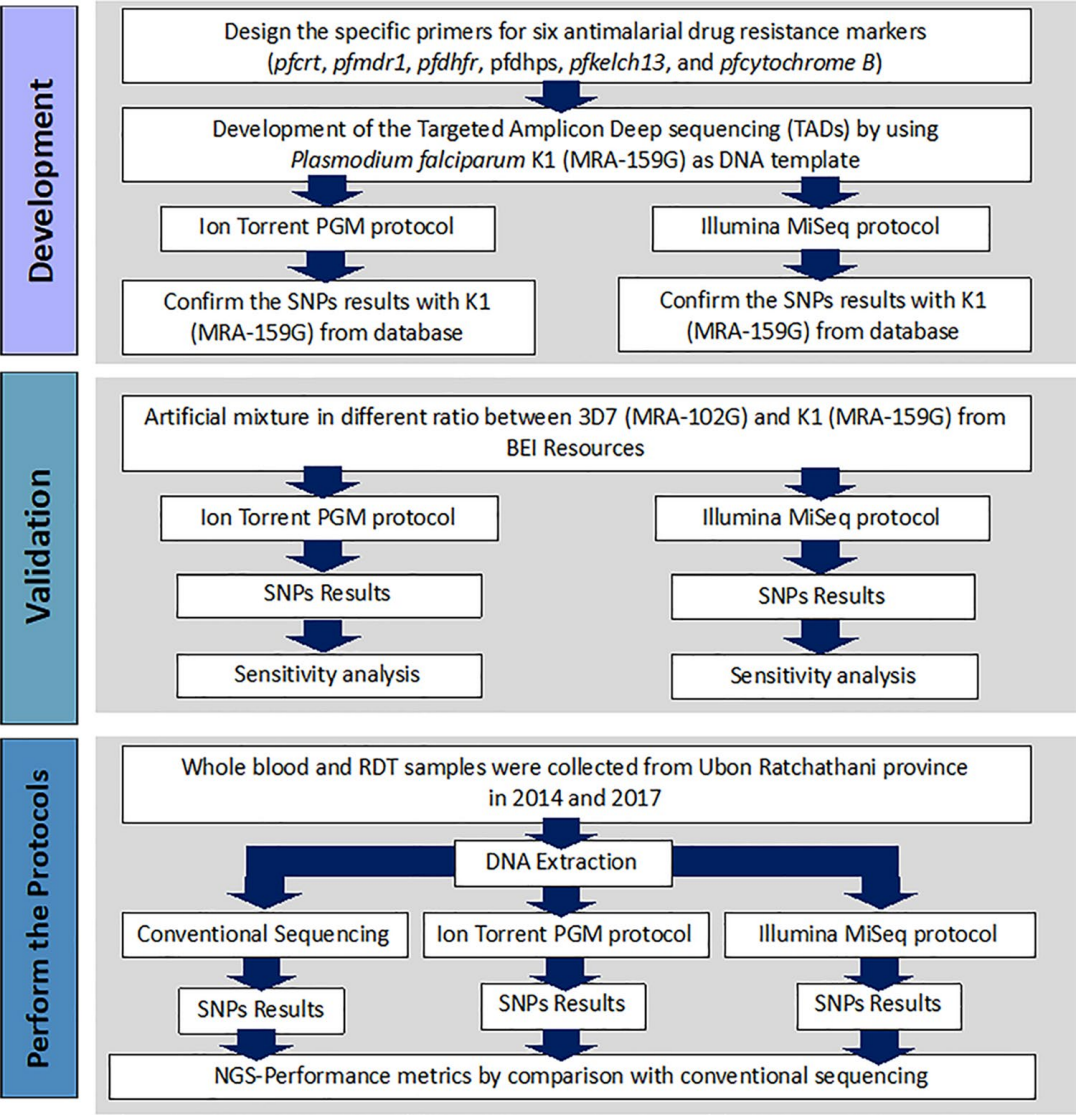
The workflow of the development of Targeted Amplicon Deep sequencing (TADs) for six drug resistance genes of *P. falciparum* by using Ion Torrent PGM and Illumina MiSeq protocol. There are three steps. Firstly, two TADs protocols were developed by using reference stain K1. Secondly, two TADs protocols were validated with *P. falciparum* strain K1 used as a positive control. Finally, two TADs protocols and conventional sequencing were performed in *P. falciparum* isolates from Ubon Ratchathani province and the SNPs results were compared.

The different NGS genotyping platforms have not been compared in detail. We here report a comparative study of Targeted Amplicon Deep sequencing (TADs) on two different NGS platforms, Ion Torrent PGM and Illumina MiSeq. We developed and validated protocols for the assessment of *P. falciparum* drug resistance markers for the two platforms and assessed the concordance of results with a reference method, Sanger sequencing by capillary electrophoresis (Fig. 1).

## Results

### Development of TADs using ion torrent PGM protocol for *P. falciparum* drug resistance markers

Target amplicons were produced from 20 whole blood samples, 5 RDT samples, and 6 artificial mixture samples containing a mix of 3D7 (reference) and K1 strain genomic DNA. The amplicons were pooled across each sample. The 25 *P. falciparum* isolates were pooled together in sequencing run 1, and the 6 artificial mixture samples were duplicated in each dilution and pooled together in sequencing run 2. The Ion Sphere Particle (ISP) loading density (percentage of wells loaded with ISPs) was 48% in sequencing run 1 and 66% in sequencing run 2. The base quality score, measuring reads which reach a quality score of Q20 or above in each run, were 1.98 M reads in run 1 and 2.85 M reads in run 2; 98.93% (1.96 M reads) in run 1 and 99.24% (2.83 M reads) in run 2 of the total reads could be aligned to the 3D7 reference genome assembly (assembly accession no. GCF_000002765.5) (Table S3). Reads from both sequencing runs were combined for further analysis. The mean number of sequences reads per amplicon was 1754 reads (minimum coverage 15 reads, maximum coverage 6,456 reads) (Fig. S1).

### Development of TADs using Illumina MiSeq protocol for *P. falciparum* drug resistance markers

Target amplicons were amplified for the six drug resistance genes in *P. falciparum* using the same samples as those used to generate the library for the Ion Torrent PGM. This sequencing run generated 13.96 M reads with 582 K/mm^2^ flow cell cluster density and 94.5% clusters passing filtering. 94.00% of sequence reads (Quality score more than Q30) could be aligned to *P. falciparum* strain 3D7. The mean number of sequencing reads per amplicon was 28,886 reads (minimum coverage 5,288 reads, maximum coverage 32,597 reads) (Fig. S2).

### Validation of two TADs protocols for *P. falciparum* drug resistance markers

**Figure 2:**
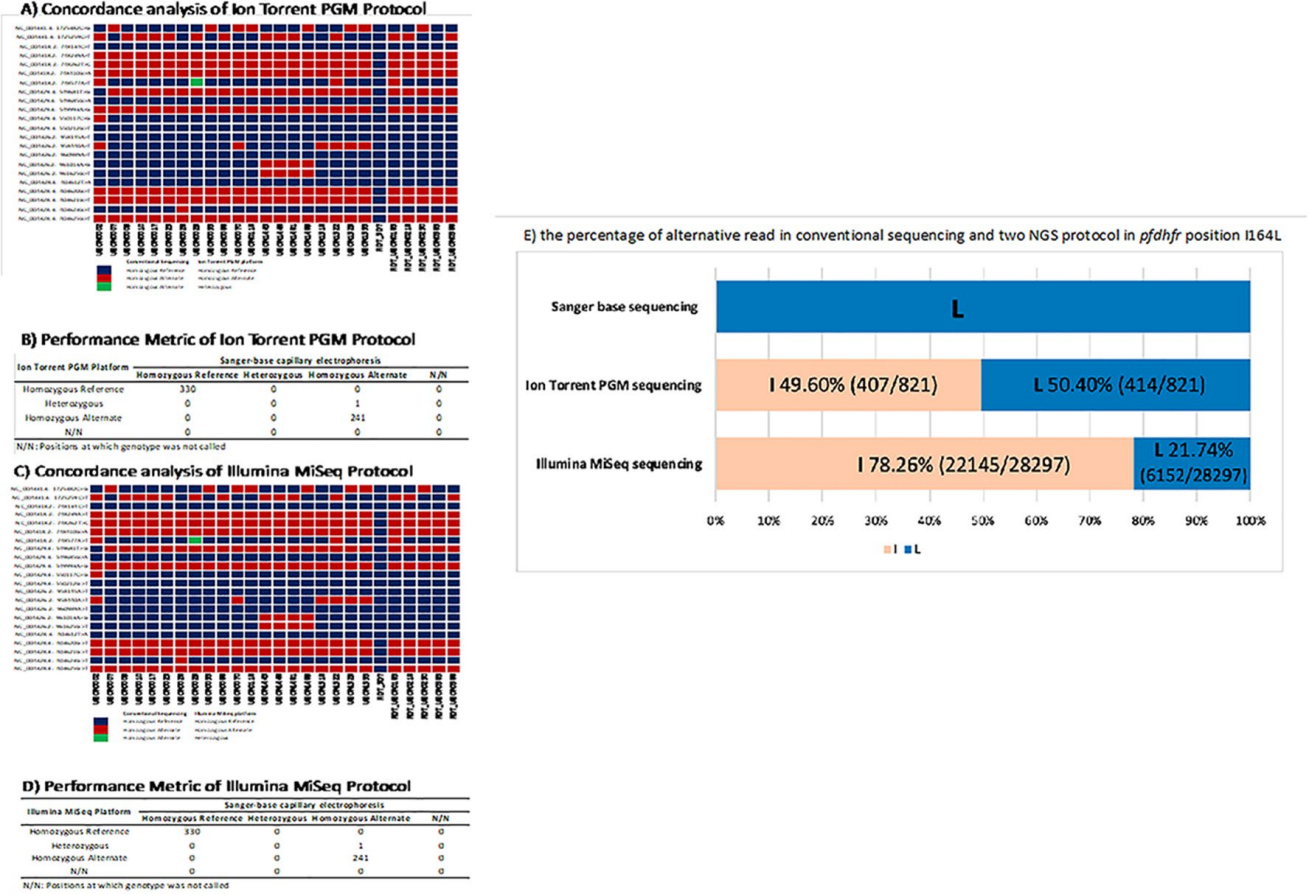
The performance metric of two TADs protocols for six drug resistance genes was calculated by conventional Sanger sequencing with capillary electrophoresis as the standard protocol. (**A**) The concordance of the mutations detected by Ion Torrent PGM protocol with Sanger sequencing. (**B**) The performance metrics results comparing the performance of Ion Torrent PGM protocol with conventional sequencing. (**C**) The concordance of the mutations detected by Illumina MiSeq protocol with Sanger sequencing. (**D**) The performance metrics results comparing the performance of Illumina MiSeq protocol with conventional sequencing. (**E**) Estimated percentage of mixture allele in *pfdhfr* gene at position I164L detected by Sanger base sequencing and two TADs protocols.

The variant calling results of the two TADs protocols were validated against variant calls produced by Sanger sequencing as the reference gold-standard. 572 SNPs of six drug resistance markers called using Sanger sequencing were compared to the variant calling results in each protocol (Tables S5 and S6) and used to calculate the sequencing accuracy, variant accuracy, false positive rate, and false negative rate, which were, respectively, 99.83% (571/572), 99.59% (241/242), 0.00% (0/242), and 0.00% (0/242) for both of the NGS protocols (Fig. 2). The one position of variant discrepancy between the two NGS platforms compared to the Sanger sequencing was one SNP in the *pfdhfr* gene at position I164L (chromosome 4 748,577 A > T) in the UBON0029 sample. This position was called as T by Sanger sequencing, but an A/T heterozygote on both NGS platforms. However, the minor allele frequency (nucleotide T) was assessed as 50.4% (414/821 reads) with Ion Torrent and as 21.74% (6152/28,297 reads) with Illumina MiSeq (Fig. 2).

### Alternative allele detection

The number of sequence reads and percentage of alternative allele at the mutation positions of artificial mixtures of 3D7 and K1 strains were calculated. Both Ion Torrent and Illumina MiSeq protocols were able to detect as low as 1% density of the alternative allele with 500X coverage with no difference in the coefficient of variant (Table 1). The average frequency of alternative sequence reads in a 1:99 mixture was 0.78% (0.98–0.58%, CV: 0.18) with Ion Torrent, and 1.02% (1.55–0.75%, CV: 0.32) with Illumina MiSeq (Table S2 and Fig. S3).

**Table 1:** Detection of mixed alleles in different artificial mixture ratios of *P. falciparum* 3D7 and K1.

Ratio in mixtures 3D7:K1 (% alternative)	Ion torrent PGM				Illumina MiSeq			
	% K1	CV	Mean K1 coverage	Mean total reads	% K1	CV	Mean K1 coverage	Mean total reads
0:100	100.00	0.00	521.40	521.40	100.00	0.00	520.40	520.40
25:75	68.06	0.03	350.40	514.20	66.38	0.08	340.00	513.00
50:50	45.06	0.14	232.60	516.20	43.36	0.09	207.80	483.40
75:25	24.03	0.10	121.60	506.20	21.84	0.12	104.40	474.60
90:10	7.41	0.13	38.00	512.00	8.42	0.18	40.80	489.40
99:1	0.78	0.18	4.20	513.00	1.02	0.32	4.80	475.00

The percentage of K1 allele was calculated from five SNP positions (the coverage reads more than 500X in two TADs protocols) with triplicate sets of random subreads. The two NGS protocols can detect down to 1.00% of K1 strain. The coefficient of variant (CV) was calculated. The CV of 1.00% K1 strain mixture in the Ion Torrent PGM protocol was 0.18 and 0.32 in the Illumina MiSeq.

### Prevalence of resistance markers in *P. falciparum* from Ubon

The antimalarial drug resistant genes of *P. falciparum, pfkelch*, *pfdhfr*, *pfdhps*, *pfcrt*, *pfmdr, and pfcytochrome B,* were assessed by Sanger sequencing and the two TADs protocols in 17 isolates (12 whole blood samples and 5 RDT samples) from 2014 and 8 isolate of whole blood samples from 2017 collected from patients in Ubon Ratchatani. All 3 sequencing methods gave identical results with no discrepancies (Tables S5 and S6).

**Figure 3:**
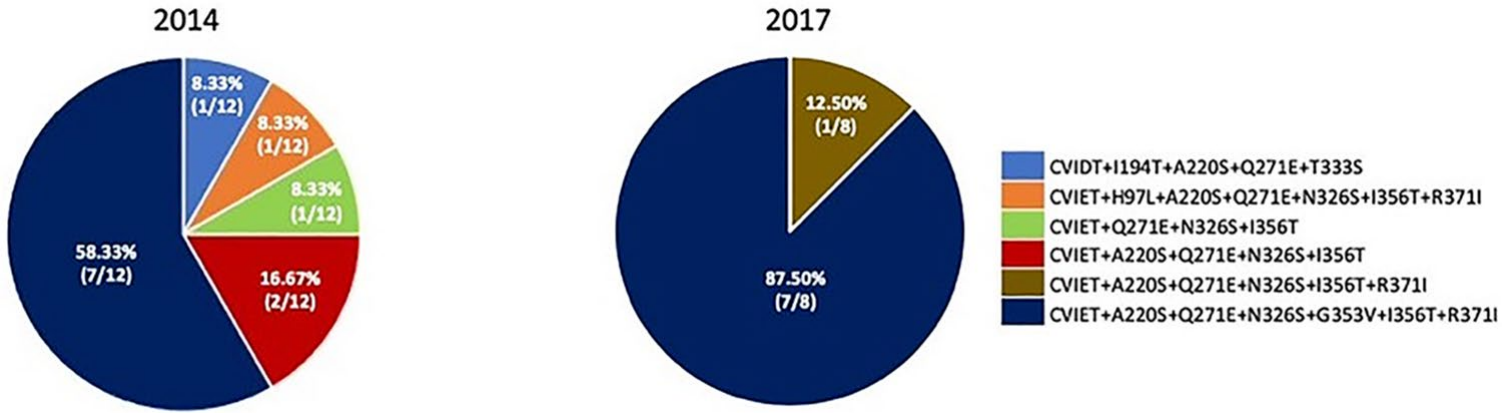
Pie charts representing haplotypes of *pfcrt* gene were observed in 12 *P. falciparum* isolates from Ubon Ratchathani in 2014 and 8 *P. falciparum* isolates from Ubon Ratchathani in 2017.

In the 2014 samples the major haplotypes of *pfcrt,* were M74I + N75E + K76T + A220S + Q271E + N326S + G353V + I356T + R371I (58%, 7/12 samples) and three less frequent haplotypes was M74I + N75E + K76T + I194T + A220S + Q271E + T333S (8.33%, 1/12 samples), M74I + N75E + K76T + H96L + A220S + Q271E + N326S + I356T + R371I(8.33%, 1/12 samples), and M74I + N75E + K76T + Q271E + N326S + I356T (8.33%, 1/12 samples). In 2017, the M74I + N75E + K76T + A220S + Q271E + N326S + G353V + I356T + R371I haplotype was dominant and was found in 88% (7/8) of samples and the less frequent haplotype was M74I + N75E + K76T A220S + Q271E + N326S + I356T + R371I (12.50%, 1/8 samples). Both these haplotypes are associated with chloroquine or piperaquine resistance (Fig. 3).

**Figure 4:**
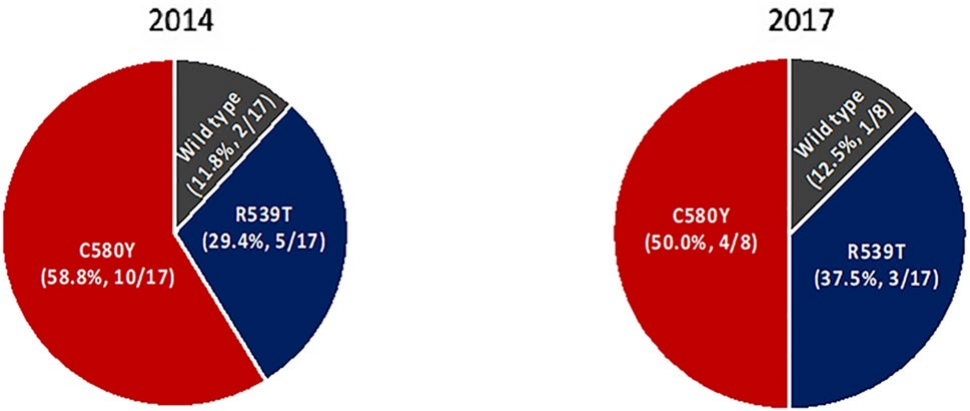
Pie charts representing proportions of mutations of *pfkelch* gene which were observed in 12 *P. falciparum* isolates from Ubon Ratchathani in 2014 and 8 *P. falciparum* isolates from Ubon Ratchathani in 2017.

Regarding *pfkelch,* in 2014, 88% (15/17) samples had mutations in the *pfkelch* gene, including the C580Y (59%, 10/17) and R539T (29%, 5/17) mutations. In 2017, 88% (7/8) samples showed a *pfkelch* mutation, including C580Y (50%, 4/8) and R539T (38%, 3/8) (Fig. 4).

For *pfdhfr*, the prevalence of *pfdhfr* quadruple mutants (mutations at N51I, C59R, S108N, and I164L) was 18% (3/17) and the prevalence of triple mutants (mutations at N51I, C59R, and S108N) was 82% (14/17) in 2014. One quadruple mutant sample showed a heterozygous SNP call at I164L/I using both NGS platforms. In 2017 the prevalence of quadruple mutants was 13% (1/8) and of triple mutants was 88% (7/8).

For *pfdhps*, the prevalence of triple mutations at S436A, A437G, and K540E was 94% (16/17) and the prevalence of triple mutations at A437G, K540E, and A581G was 6% (1/17) in 2014. In 2017, all 7/7 samples showed the triple mutations at S436A, A437G, and K540E.

For *pfmdr1*, the prevalence of double mutants with haplotype Y184F, N1042D and F1226F was 12% (2/17), and of single mutants with haplotype Y184Y, N1042D and F1226F was 88% (15/17) in 2014. In 2017, the prevalence of triple mutants with haplotype Y184F, N1042D, and F1226Y of *pfmdr1* was 50% (4/8) and of the single mutant with haplotype Y184Y, N1042D and F1226F was 50% (4/8).

**Figure 5:**
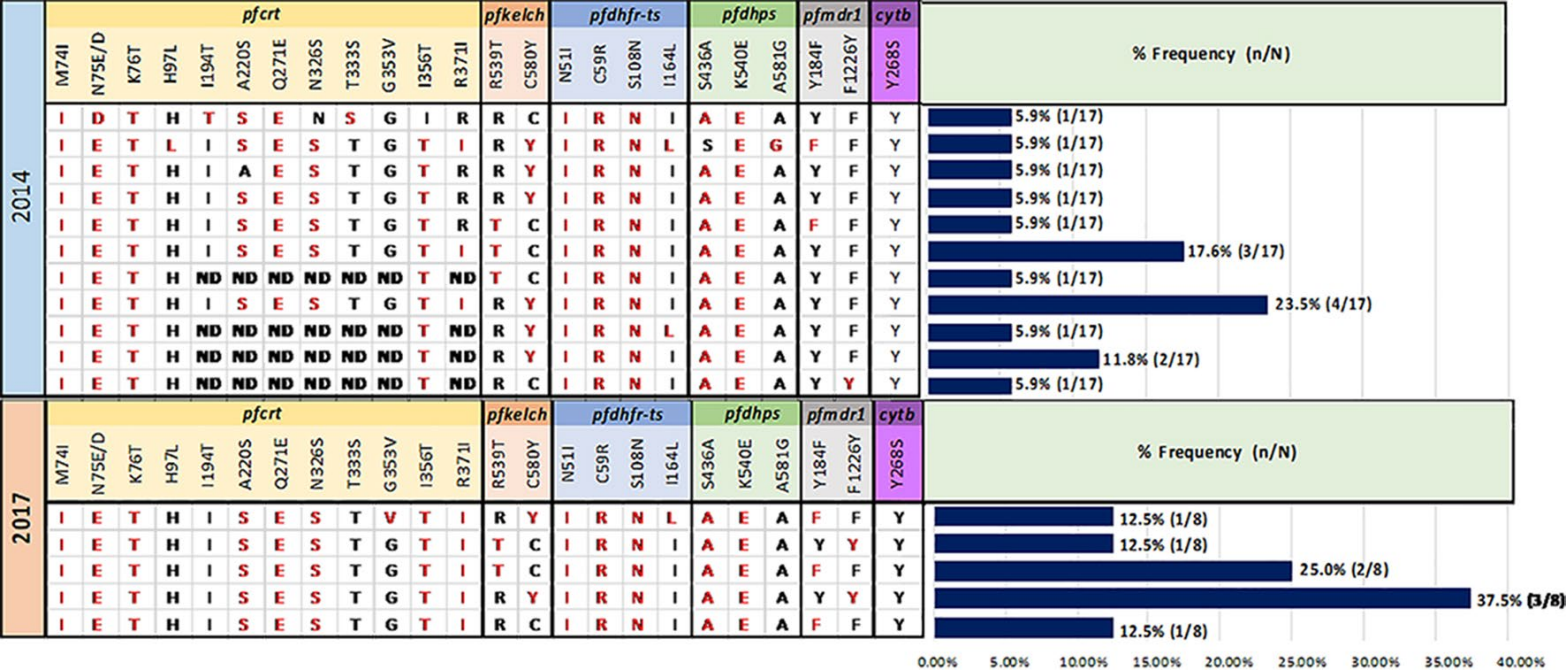
Bar chart represents the genetic patterns which were observed in six drug resistance genes in Ubon Ratchathani province in 2014 and 2017. The results of the two TADs protocols are not different from conventional Sanger sequencing. In the Ubon0029 isolate, *pfdhfr* gene at position I164L was detected as a mixed allele by two TADs protocols, but conventional Sanger sequencing called it a homozygous mutation.

Considering combinations of six antimalarial drug resistance markers, the most prevalent haplotype in Ubon Ratchathani in 2014 was M74I + N75E + K76T + A220S + Q271E + N326S + I356T + R371I (*pfcrt*), C580Y (*pfkelch*), N51I + C59R + S108N (*pfdhfr*), S436A + K540E (*pfdhps*), WT (*pfmdr1*), and WT at position Y268S (*pfcytochrome b*), which was present in 24% (4/17) of samples. In 2017 the most prevalent haplotype was the same as in 2014 but with *pfmdr1* F1226Y instead of WT and observed in 36% (3/8) of samples (Fig. 5).

## Discussion

Targeted Amplicon Deep sequencing (TADs) is a next generation sequencing technique amplifying and sequencing targeted regions of the genome. The technique has been developed and optimized for the study of various pathogens, including *Mycobacterium tuberculosis*^14^, HIV^15^, and SARS-CoV-2^16^, as well as *P. falciparum* where TADs have been developed for assessing parasite population diversity^17–24^ and antimalarial drug resistance markers^2–4,6–8,25^. Sanger sequencing can also more accurately identify high frequency alleles.

In our study, NGS protocols were successfully developed for two widely used NGS platforms. We found that the number of total reads, and therefore the total number of reads mapped to the reference genome was higher with Illumina MiSeq compared to Ion Torrent PGM. The average number of sequence reads per amplicon was also higher using Illumina MiSeq than Ion Torrent PGM. These results are consistent with previous studies^2,3,7^ and can be explained by the higher capacity of Illumina MiSeq sequencing.

Both TADs methods using our NGS protocols provided highly accurate SNP calls. The *P. falciparum* SNPs in the K1 strain called from two TADs protocols were in complete agreement with the reference method. One minor allele variant in a ‘heterozygous’ mixed sample was identified correctly by the NGS method, but not picked up by Sanger sequencing, which can only identify ‘homozygous’ variants^7^.

Using our TADs protocols, both NGS platform showed a high sensitivity to detect minor alleles in artificially mixed *P. falciparum* strains. With 500X coverage of each of the 5 SNPs positions known to differ between the two assessed strains, including C59R, S108N (*pfdhfr*), A581G (*pfdhps*), N86Y (*pfmdr1*), and R371I (*pfcrt*), both platforms could detect the alternative allele down to a relative density of only 1% or 0.1 ng/µL. This is important for antimalarial drug resistance surveillance, in particular in areas with higher transmission, where multiclonal infections will be common, and drug resistant minor clones can be easily missed. A recent study reported that for *P. falciparum* 10,000 reads were sufficient to detect a minor allele at a ratio of 1:1000 using TADs^26^. However, detection accuracy will depend on the method, as other studies showed that a minor allele population of 9% frequency was detected with 692 mean reads^27^, and at 5% frequency with 250 mean reads^28^. Our protocol described in this study enables the detection of a minor allele at 1% frequency with 500 reads. However, a minority of the SNPs sequenced using TADs in this study had read coverage of 500 reads or more, allowing us to assess only 12 positions in this study. The capacity to detect alleles present at very low frequencies could require high sequencing coverage, leading to a trade-off between detection of low frequency alleles and cost-effective surveillance. However, this is a key strength of the NGS approach, as Sanger sequencing, while accurate with high-frequency alleles, cannot detect these low frequency alleles.

Although not the primary aim of this study, our results showed persistence in Ubon Ratchathani of the *pfcrt* M74I + N75E + K76T + A220S + Q271E + N326S + G353V + I356T + R371I haplotype at 58% (7/12) in 2014 and 88% (7/8) in 2017. This might be related to the continued drug pressure on the parasite population by chloroquine, which is still used for the treatment of vivax malaria. The *pfcrt* mutation at position 353 has been associated with piperaquine resistance, in addition to the well described marker, *Plasmepsin2* amplification^29,30^. Our study also showed persistence of mutations in the propeller region of the *pfkelch* gene, strongly associated with artemisinin resistance *pfkelch*^31^ and confirmed by other studies from the same region^32–35^. Since our study, the first-line drug for the treatment of uncomplicated falciparum malaria in Eastern Thailand was changed from dihydroartemisinin-piperaquine to artesunate-pyronaridine. Continued surveillance for drug resistance will remain important as changes in treatment alter the selective pressure on different resistance variants.

NGS is cheaper compared to conventional methods. In our study the total cost of consumables to sequence six drug resistance genes per sample was $62.72 for the Ion Torrent PGM platform and $60.87 for Illumina MiSeq when multiplexing 96 samples per sequencing run. This compared to a cost of $450.90 using conventional Sanger sequencing). The NGS platforms, particularly the MiSeq, allow for more scalability to further reduce costs as samples can be multiplexed at higher numbers. Moreover, using our protocol cost and time were reduced by using multiplex PCR of targeted amplicons by selecting primer pairs with the same annealing temperature and similar size, and dividing the amplicons into two multiplex pools to ensure they are not overlapping. The amplicons could be designed to give different PCR product sizes to allow for extraction of the gel bands. An alternative could be to skip the gel extraction step, thus further reducing costs and time. The Ion Torrent PGM protocol we developed used 18 targeted PCR reactions with similar annealing temperatures which could be run as two multiplex pools, whereas the Illumina MiSeq protocol used 17 targeted PCR reactions also run as two multiplex pools. Costs can be reduced further by amplifying only known drug resistance marker regions rather than complete gene sequences. It should be recognized, though, that this precludes detecting novel drug resistance mutations falling outside these regions. A targeted approach translates to 26 targeted PCR reactions for the Ion Torrent PGM platform and 16 targeted PCR reactions for llumina MiSeq.

In summary, we developed two Targeted Amplicon Deep sequencing (TADs) protocols for genotyping of *P. falciparum* drug resistant markers, targeting the full length of five drug resistant genes (*pfmdr1*, *pfkelch*, *pfcrt*, *pfdhfr*, and *pfdhps*) and *pfcytochrome b* (position 268 only) using the Ion Torrent PGM and Illumina MiSeq platforms. The cost per sample was slightly lower with Illumina MiSeq compared to Ion Torrent PGM, but the latter method does not require bioinformatics expertise for data analysis. There are several advantages of these platforms compared to Sanger sequencing with capillary electrophoresis, which is not suitable for high throughput analysis, is labor-intensive and expensive. We conclude that both platforms using the newly developed TADs protocols are suitable for the surveillance of *P. falciparum* drug resistance markers in malaria endemic areas.

## Methods

### Genomic DNA and samples collection

We studied three types of samples (Fig. 1):

1. Genomic DNA of *P. falciparum* strains 3D7 (MRA -102G) and K1 (MRA-159G) from MR4-BEI Resource was used as a positive control, and the two strains were mixed in 6 different proportions to investigate minor allele detection.
2. Whole blood samples were collected from Ubon Ratchathani province, Thailand, in 2014 (N = 12) and 2017 (N = 8) collected during outbreaks of artemisinin resistance *P. falciparum* malaria. Geometric mean parasite densities were 118,749.09 parasites/uL (High-Power Field: HPF Method). The gDNA was extracted from whole blood samples using the QIAamp® DNA Mini Kit (Qiagen, CA, USA), according to the manufacturer’s instructions.
3. Malaria P.f/Pan antigen test (RDT) samples were performed by using whole blood samples (N = 5) from Ubon Ratchathani provide in 2014. Geometric mean parasite densities were 125,600 parasites/uL. The gDNA was extracted by boiling protocol^36^.

Ethical approval for the study was obtained from the ethical review board of the Faculty of Tropical Medicine, Mahidol University (TMEC 20-36).

### Conventional sanger sequencing

The full-length sequences of *pfkelch, multidrug resistance transporter* (*pfmdr1*), *chloroquine resistance transporter* (*pfcrt*), *dihydrofolate* (*pfdhfr*), *dihydropteroate synthase* (*pfdhps*) and *pfcytochrom b* mutation at the amino acid position Y268 were obtained by nested PCR amplification using previously described primer^37–39^ following conventional sequencing by ABI Sequencer (Macrogen Inc, South Korea). The mutations were analyzed using the BioEdit program (version 7.2; https://bioedit.software.informer.com/7.2/) (Tom Hall, North Carolina State University).

### Development of ion torrent PGM protocol

#### Primer design

70 oligonucleotide primer pairs (see Table S1 in the supplemental material) for six antimalarial drug resistance genes, *pfkelch*, *pfcrt*, *pfdhfr*, *pfdhps*, *pfmdr1*, and *pfcytochrome b*, were designed by Primer3 program (version 4.1.0; https://primer3.ut.ee)^40,41^ using 3D7 reference genome from NCBI database. Primer-Blast (https://www.ncbi.nlm.nih.gov/tools/primer-blast/) on NCBI data was used to improve specificity of primers.

#### Targeted amplicon amplification

Singleplex PCR was performed separately on all samples and control mixtures to amplify the targeted amplicons of six antimalarial drug resistance genes with Q5 High-Fidelity DNA polymerase (New England BioLabs, MA, USA). The PCR products were purified and concentrated using FavorPrep GEL/PCR Purification Mini Kit. The purified PCR products were quantified and improved the purity by Nanodrop (Thermo Fisher Scientific, USA). The concentration of purified PCR products were adjusted to 7.6 ng/ul, and then the PCR amplicons were pooled.

#### Library preparation and PGM sequencing

The pooled amplicons of each sample were ligated with barcode and adapter by Ion Plus Fragment Library kit (Thermo Fisher Scientific, USA). The library was size selected by Agencourt AMPure XPbeads (Beckman Coulter, CA, USA) and amplified with Platinum PCR superMix High fidelity (Thermo Fisher Scientific, USA). Libraries were normalized and pooled at 100 pM. Emulsion PCR was performed on the pooled DNA library by Ion PGM Hi-Q View ISP kit on the Ion OneTouch 2 system (Thermo Fisher Scientific, USA). The positive Ion Sphere particles were enriched on OneTouch ES, loaded onto Ion316 Chip kit V2 and sequenced on PGM sequencer. Two library pools PF-RUN001 and PF-RUN002, were loaded into each 316-v2 chip.

#### Variant calling and annotation

Sequencing data were demultiplexed and quality filtered by BaseCaller program (https://tools.epigenetic.ru/ts-doc/ion-docs/GUID-5343E87B-C50A-4AEC-8C36-A189C352E5C1.html). The sequence reads were aligned to *P. falciparum* 3D7 genome by Torrent Mapping Alignment Program (version 5.0; https://github.com/iontorrent/TMAP). The sequence reads across the amplicon were investigated by Coverage Analysis plugin (version 5.0.28; https://ionreporter.thermofisher.com/ionreporter/help/GUID-7355C2DB-5AC9-4EF6-B166-1F55ABE0F1BA.html). then, the variant data were filtered by custom criteria: variant quality score was greater than 10, the variant was supported by both forward and reverse sequence reads, the minimum frequency of variant is more than 2% and the minimum coverage of variant is more than 10 reads. Custom criteria were not applied for variant calling for the artificial mixture models.

### Development of Illumina MiSeq protocol

#### Primer design

46 oligonucleotide primer pairs (see Table S1 in the supplemental material) of six antimalarial drug resistance genes, *pfkelch*, *pfcrt*, *pfdhfr*, *pfdhps*, *pfmdr1*, and *pfcytochrome b*, were designed by Primer3 program (version 4.1.0; https://primer3.ut.ee) based on the principles described in 16S Metagenomic Sequencing Library Preparation protocol (Illumina, USA). Primer-Blast (https://www.ncbi.nlm.nih.gov/tools/primer-blast/) on NCBI data was used to improve specificity of primers. Multiple Primer Analyzer program (https://www.thermofisher.com/th/en/home/brands/thermo-scientific/molecular-biology/molecular-biology-learning-center/molecular-biology-resource-library/thermo-scientific-web-tools/multiple-primer-analyzer.html) (Thermo Fisher Scientific, USA) was used to predict homo- and heterodimer.

#### Targeted amplicon amplification

Singleplex PCR was performed separately on all samples and control mixtures to amplify the targeted amplicon of six antimalarial drug resistance genes with Q5 High-Fidelity DNA polymerase (New England BioLabs, MA, USA). The PCR products were purified using FavorPrep GEL/PCR Purification Mini Kit and quantified by Nanodrop (Thermo Fisher Scientific, USA). The concentration of purified PCR product was adjusted to 33 nM, and then the PCR amplicons were pooled.

#### Library preparation and MiSeq sequencing

Illumina Nextera XT Index kit (Illumina, USA) was used to ligate indexes to the pooled PCR amplicons. The pooled library was assessed using Bioanalyzer DNA 1000 Chip (Agilent Technology, USA) and measured by Qubit (Thermo Fisher Scientific, USA). The PCR library was normalized at 4 nM, and then each library was pooled at 5 ul/sample. 8 pM of denatured library was loaded onto the sequencer, and Phix control was included at 5%. MiSeq v3 Reagent Kit v3 600-cycle (Illumina, USA) was used for sequencing.

#### Variant calling and annotation

The quality of the sequence run was monitored by Sequencing Analysis Viewer (SAV) (version 2.4.5; https://support.illumina.com/sequencing/sequencing_software/sequencing_analysis_viewer_sav/downloads.html) (Illumina, USA). Raw data were demultiplexed and generated FastQ files using standard quality filtering parameters by the MiSeq Reporter (MSR) (version 2.6.2.3; https://www.illumina.com/systems/sequencing-platforms/miseq/products-services/miseq-reporter.html) (Illumina, USA). FastQ files were filtered for quality with the FASTQ toolkit (version 2.2.0; https://www.illumina.com/products/by-type/informatics-products/basespace-sequence-hub/apps/fastq-toolkit.html)^42^ and mapped to the *P. falciparum* 3D7 reference genome with Burrows-Wheeler Aligner (BWA) (version 0.7.17-r1188; https://sourceforge.net/p/bio-bwa/mailman/bio-bwa-help/?limit=50). BWA generated alignment file in BAM format. Variant calling was performed using the SAMtools (version 0.1.16; https://sourceforge.net/projects/samtools/files/samtools/0.1.16/) mpileup command^43^. The variant data were filtered by custom criteria which similar to Ion Torrent PGM: the variant quality score was greater than 10, the variant was supported by both forward and reverse sequence reads, the minimum frequency of variant is more than 2% and the minimum coverage of variant is more than 10 reads. SNPs were annotated using *P. falciparum* 3D7 as reference by IGV program (version 2.3; https://software.broadinstitute.org/software/igv/)^44^. Custom criteria were not applied for variant calling for the artificial mixture models.

### Alternative allele detection

The alternative allele detection of the two NGS platforms was calculated by using an artificial mixture of two strains. Two strains artificial mixture were performed by mixing 10 ng/ul parasite genomic DNA of *P. falciparum* strains 3D7 (MRA -102G) and K1 (MRA-159G) in different ratio of volume with the same standard genomic concentration from BEI Resources (https://www.beiresources.org/Home.aspx). The ratios used were 0:100 (3D7 0 ul: K1 100 ul), 25:75 (3D7 25 ul: K1 75 ul), 50:50 (3D7 50 ul: K1 50 ul), 75:25 (3D7 75 ul: K1 25 ul), 90:10 (3D7 90 ul: K1 10 ul), and 99:1(3D7 99 ul: K1 1 ul). The final concentration of all mixtures was 10 ng/ul. We used the same artificial strain mixtures to perform PCR amplification and sequencing on both NGS platforms. The custom criteria not apply in variant calling step. K1 and 3D7 have different alleles at 12 positions in six drug resistance genes. Due to the difference in read coverage between platforms and amplicons, the coverage of sequence reads was downsampled to give normalized 500X coverage by using Lander/Waterman equation. The Ion Torrent PGM platform were calculated as single end sequencing reads, and Illumina MiSeq platform were calculated as paired end sequencing reads. Only 5 mutation positions, consisting of *pfdhfr* at position C59R (NC_004318.2:748262 T > C), *pfdhfr* at position S108N (NC_004318.2: 748410G > A), *pfdhps* at position A581G (NC_0043, 29.3:550117C > G), *pfmdr1* at position N86Y (NC_0043262.2: 958145A > T), and *pfcrt* at position R371I (NC_004328.3: 405838G > T), had coverage more than 500X and were used in alternative allele detection, respectively. Samtools view program (version 1.9; https://sourceforge.net/projects/samtools/files/samtools/1.9/) was used to perform subsampling of reads at the mutation position with triplicate random subsamples. IGV program (version 2.3; https://software.broadinstitute.org/software/igv/) was used to visualize the sequence reads.

All methods were carried out in accordance with relevant guidelines and regulations.

### Cost calculation

The cost was calculated per sample, with cost including consumable plasticware, reagents from DNA extraction step to sequencing step, and primer for each sequencing platform (conventional sequencing: ABI 3031XL genetic analyser, Ion Torrent PGM, and Illumina Miseq).

### Ethics approval and consent to participate

Ethical approvals for the study were obtained from the ethical review committees of the Faculty of Tropical Medicine, Mahidol University (MUTM2020-044–01). Informed consent was obtained from all participants.

## Supplementary Information

Supplementary Information 1.

Supplementary Information 2.

## Data Availability

The sequencing data in this study are publicly available in NCBI SRA: PRJNA773623.
