# Supplementary material for: Comparative analysis of targeted next-generation sequencing for Plasmodium falciparum drug resistance markers
Source: Sci Rep. 2022 Apr 1;12:5563. doi: 10.1038/s41598-022-09474-5 (PMC8974807; doi:10.1038/s41598-022-09474-5)
Supplement: Supplementary file 2 — Supplementary Information 2. [file 41598_2022_9474_MOESM2_ESM.pdf]

Supplementary data

Comparative analysis of targeted next-generation sequencing for *Plasmodium falciparum* drug resistance markers

Table S1 Primers used for six *P. falciparum* drug resistance markers in Ion torrent PGM protocol

Excel file name: Supplement_Table S1 Primers used for six P. falciparum drug resistance markers in both of NGS protocol

Table S2 Primers used for six *P. falciparum* drug resistance markers in Illumina MiSeq protocol

Excel file name: Supplement_Table S2 Primers used for six P. falciparum drug resistance markers in Illumina MiSeq

Metric	Factors	% (Reads/Total Reads) in PF-RUN001	% (Reads/Total Reads) in PF-RUN002
Base Quality Metric	Percentage of test fragment (sequencing control) reads	**1.04%** (22,046/3,026,446)	**0.64%** (26,928/4,197,923)
	Percentage of live library	**98.11%** (2,969,279/3,026,446)	**98.99%** (4,155,503/4,197,923)
	Percentage of polyclonal	**26.76%** (794,466/2,969,279)	**25.53%** (1,061,105/4,155,503)
	Percentage of low quality reads	**4.38%** (130,187/2,969,279)	**3.51%** (145,846/4,155,503)
	Percentage of adapter dimer	**0.02%** (483/2,969,279)	**0.02%** (1,023/4,155,503)
	Final library Ion Sphere Particles (ISPs)	**68.84%** (2,044,143/2,969,279)	**70.93%** (2,947,529/4,155,503)
	The mean of read length	265 bp	265 bp
Mapping Metric	Percentage of total reads	**100.00%** (1,985,955/1,985,955)	**100.00%** (2,856,295/2,856,295)
	Percentage of reads aligned to reference	**98.93%** (1,964,658/1,985,955)	**99.24%** (2,834,508/2,856,295)
	Percentage of unaligned reads	**1.00%** (21,297/1,985,955)	**1.00%** (21,787/2,856,295)
	Percentage of alignment quality score more than Q20	**95.00%** (462 Mbp/486 Mbp)	**92.00%** (640 Mbp/694 Mbp)
Variant Calling Metric	Variant allele frequency	**88.00%** (Range: 14.00 – 100.00)	**86.27%** (Range: 22.80 – 100.00)
	Variant call quality score	**5649.61** (Range: 11.7 – 33382.7)	**1906.93** (Range: 21.38 – 2982.21)
	Sequence reads with the variant	**910.27** (Range: 11 – 5135)	**1245.55%** (Range: 15 – 6,456)

Table S3 The base quality metrics, mapping metrics and variant calling metrics of *P. falciparum* drug resistance markers using Ion Torrent PGM protocol.

Metric	Factors	% (Reads/Total Reads)
**Illumina MiSeq sequencing platform**		
Base Quality Metric	Percentage of Sequencing reads	**100.00%** (13,956,156/13,956,156)
	Percentage of PhiX positive control	**8.90%**
	Percentage of Passing filter reads	**95.00%** (13,196,576/13,956,156)
	Cluster Density	**582 K/mm^2**
	Percentage of Reads quality score more than 30	**73.00%** (10,132,169/13,956,156)
Mapping Metric	Percentage of total reads	**100.00%** (10,132,169/10,132,169)
	Percentage of reads aligned to reference	**94.00%** (9,559,079/10,132,169)
	Percentage of unaligned reads	**6.00%** (573,090/10,132,169)
Variant Calling Metric	Variant allele frequency	**84.45%** (20.00% – 100%)
	Variant call quality score	**71046.02** (20.50 – 254,358)

Table S4 The base quality metric, mapping metric, and variant calling metric of Illumina Miseq sequencing protocol.

Table S5 The annotation of SNPs of six drug resistance genes using Ion Torrent protocol using *P. falciparum* (Assembly accession: GCA_000002765.3) as reference genome.

Excel file name: Supplement_Table S3 The annotation of SNPs of six drug resistance genes using Ion Torrent protocol using P. falciparum as reference genome

Table S6 The annotation of SNPs of six drug resistance genes using Illumina MiSeq protocol using *P. falciparum* (Assembly accession: GCA_000002765.3) as reference genome.

Excel file name: Supplement_Table S4 The annotation of SNPs of six drug resistance genes using MiSeq protocol using P. falciparum as reference

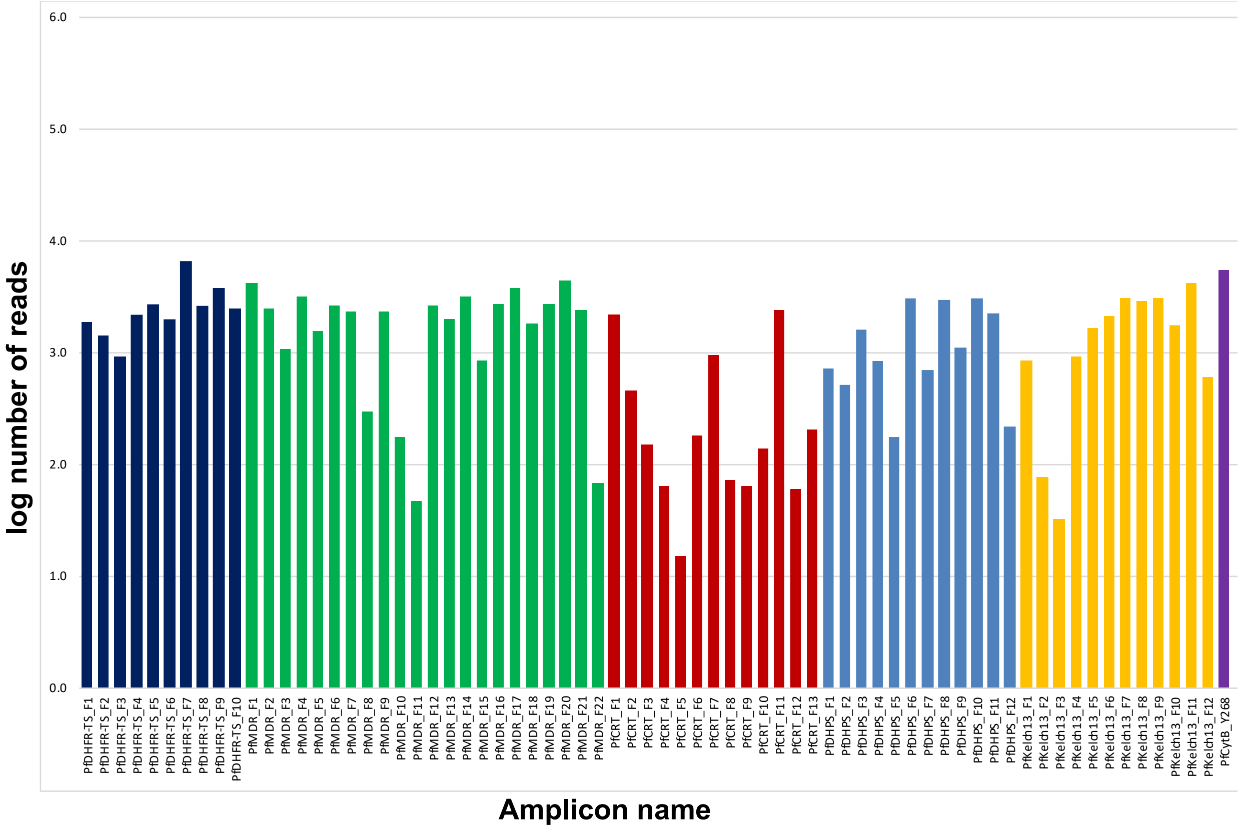

Figure S1 Bar chart representing the mean coverage reads of each targeted amplicon in *P. falciparum* using Ion Torrent PGM. X axis represents log number of reads. Y axis represent the amplicon name.

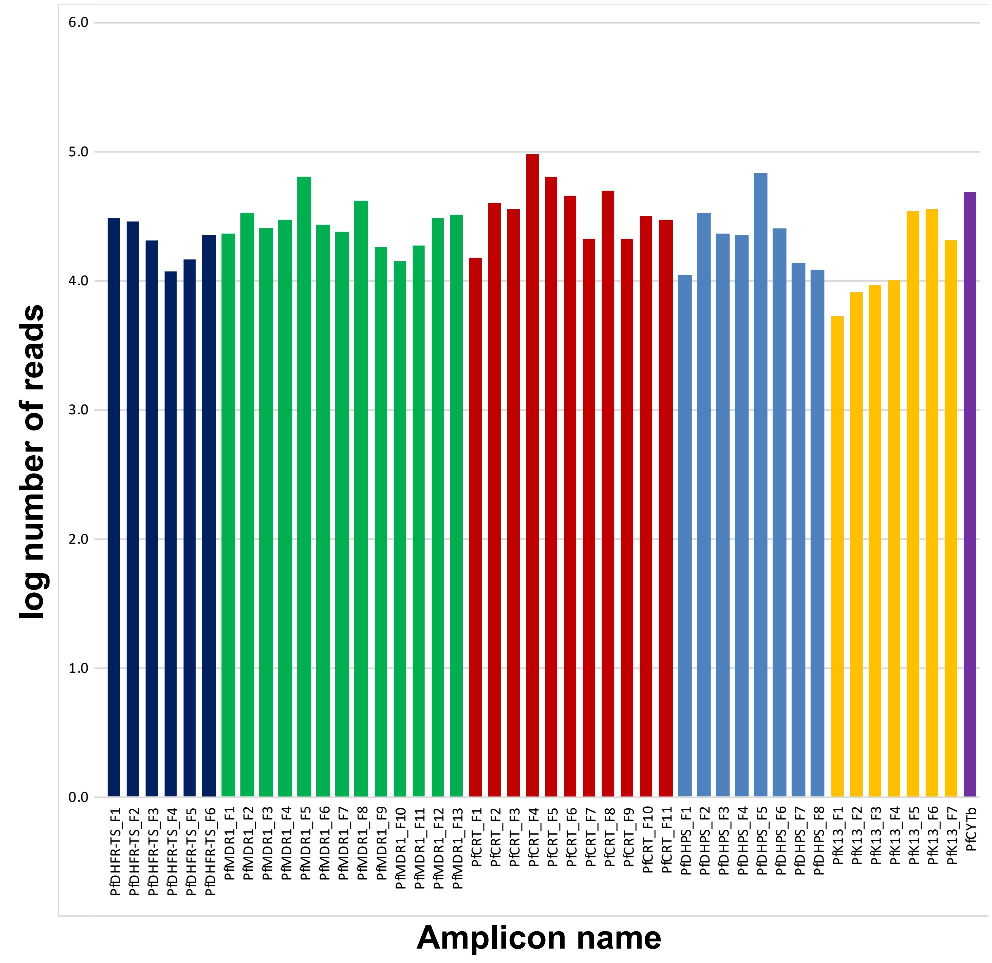

Figure S2 Bar chart representing the mean coverage reads of each targeted amplicon in *P. falciparum* using Illumina MiSeq. X axis represents the number of reads. Y axis represent the amplicon name.

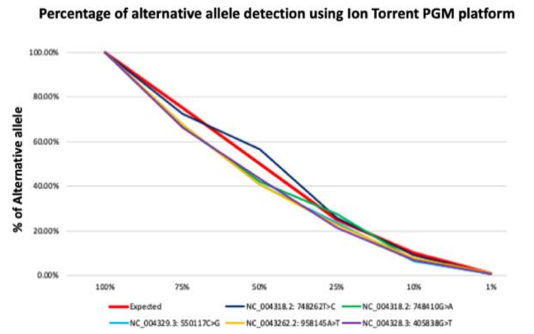

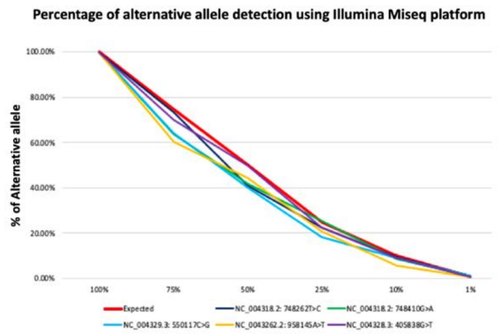

Mutation Positions	100%	75%	50%	25%	10%	1%
Expected	100.00%	75.00%	50.00%	25.00%	10.00%	1.00%
NC_004318.2: 748262T>C	100.00%	72.37%	56.31%	25.85%	8.97%	0.60%
NC_004318.2: 748410G>A	100.00%	66.79%	41.84%	27.29%	6.94%	0.62%
NC_004329.3: 550117C>G	100.00%	67.33%	40.52%	23.65%	6.57%	0.68%
NC_0043262.2: 958145A>T	100.00%	67.62%	40.80%	22.45%	7.68%	0.98%
NC_004328.3: 405838G>T	100.00%	66.03%	43.30%	21.18%	7.05%	0.74%

Mutation Positions	100%	75%	50%	25%	10%	1%
Expected	100.00%	75.00%	50.00%	25.00%	10.00%	1.00%
NC_004318.2: 748262T>C	100.00%	73.31%	40.82%	22.37%	9.46%	1.05%
NC_004318.2: 748410G>A	100.00%	63.81%	41.76%	25.45%	8.57%	0.87%
NC_004329.3: 550117C>G	100.00%	64.30%	40.28%	18.25%	9.12%	1.13%
NC_0043262.2: 958145A>T	100.00%	60.27%	44.18%	20.99%	5.74%	0.72%
NC_004328.3: 405838G>T	100.00%	70.21%	49.75%	22.31%	9.19%	0.81%

Figure S3 Line chart representing the percentage of alternative allele from artificial mixture between 3D7 and K1 in different ratio. The percentage of alternative allele were calculated from the coverage read higher than 500X by triplicate random subreads.
